# DLK1 Is Associated with Stemness Phenotype in Medullary Thyroid Carcinoma Cell Lines

**DOI:** 10.3390/ijms252211924

**Published:** 2024-11-06

**Authors:** Danilo Dias da Silva, Rodrigo Pinheiro Araldi, Mariana Rocha Belizario, Welbert Gomes Rocha, Rui Monteiro de Barros Maciel, Janete Maria Cerutti

**Affiliations:** 1 Genetic Bases of Thyroid Tumour Laboratory, Division of Genetics, Department of Morphology and Genetics, Escola Paulista de Medicina, Universidade Federal de São Paulo, São Paulo 04039-032, SP, Brazil; ddsilva@unifesp.br (D.D.d.S.); rodrigo.pinheiro.araldi@gmail.com (R.P.A.); m.belizario@unifesp.br (M.R.B.); welbert.rocha29@unifesp.br (W.G.R.); 2 Laboratório de Endocrinologia Molecular e Translacional, Disciplina de Endocrinologia e Metabologia, Departamento de Medicina, Escola Paulista de Medicina, Universidade Federal de São Paulo, São Paulo 04039-032, SP, Brazil; rui.maciel@unifesp.br

**Keywords:** medullary thyroid carcinoma, cancer stem cells, DLK1, RET, CD44, CD133, p.M918T

## Abstract

Medullary thyroid carcinoma (MTC) is a rare and aggressive tumor, often requiring systemic treatment in advanced or metastatic stages, where drug resistance presents a significant challenge. Given the role of cancer stem cells (CSCs) in cancer recurrence and drug resistance, we aimed to identify CSC subpopulations within two MTC cell lines harboring pathogenic variants in the two most common MEN2-associated codons. We analyzed 15 stemness-associated markers, along with well-established thyroid stem cell markers (CD133, CD44, and ALDH1), a novel candidate (DLK1), and multidrug resistance proteins (MRP1 and MRP3). The ability to efflux the fluorescent dye Hoechst 3342 and form spheroids, representing CSC behavior, was also assessed. MZ-CRC-1 cells (p.M918T) displayed higher expressions of canonical markers, DLK1, and MRP proteins than TT cells (p.C634W). MZ-CRC-1 cells also formed more spheroids and showed less dye accumulation (*p* < 0.0001). Finally, we observed that DLK1+ cells (those expressing DLK1) in both cell lines exhibited significantly higher levels of stemness markers compared to DLK1− cells (those lacking DLK1 expression). These findings underscore DLK1’s role in enhancing the stemness phenotype, providing valuable insights into MTC progression and resistance and suggesting potential therapeutic implications.

## 1. Introduction

Medullary thyroid carcinoma (MTC) is a rare tumor that arises from calcitonin-secreting parafollicular C-cells in the thyroid gland. Approximately 25–40% of MTC cases are hereditary, occurring as part of multiple endocrine neoplasia type 2A or 2B (MEN 2A or MEN 2B) syndromes, while the remaining 60–75% are sporadic cases [1,2].

Familial cases are attributed to germline-activating pathogenic variants in the *RET* gene, located at 10q11.21 [2]. Despite a well-established genotype–phenotype correlation between RET pathogenic variants and MEN 2 syndromes, there exists significant variability in the age of onset, disease aggressiveness, and outcomes both within and among families with the same RET pathogenic variant [1,2,3,4,5,6,7]. Additionally, somatic pathogenic variants in the *RET* gene, and less frequently in the *RAS* genes, are identified in sporadic MTCs [8,9,10].

Both hereditary and sporadic forms of MTC often present with cervical lymph node involvement at diagnosis, occurring in approximately 50% of cases. The primary treatment is extensive and meticulous surgical resection. However, distant metastases are observed in 10–15% of patients, underscoring the need for thorough assessment of disease extent and severity [1,2].

In the absence of a standardized risk-stratification system, clinical-pathological prognostic factors such as age at diagnosis, gender, tumor stage, tumor volume doubling time, regional lymph node involvement, and distant metastases are used to identify high-risk patients who may benefit from more aggressive treatment [2,11].

For patients with progressive or symptomatic metastatic disease not amenable to surgery, radiotherapy and targeted systemic therapies are effective interventions. However, despite their efficacy and generally favorable tolerability, resistance mechanisms have been documented, highlighting the need for ongoing research and drug development to overcome these challenges [12,13,14,15,16,17,18,19,20,21,22,23,24,25].

In our prior study, we discovered recurrent somatic copy number alterations (CNAs) affecting the *DLK1* locus (14q32.2) in both sporadic and hereditary cases of MTC [26]. A correlation was observed between *DLK1* CNA and pathological features indicative of more-aggressive disease and poorer outcomes (larger tumor size and advanced AJCC tumor stages).

Delta-like non-canonical Notch ligand 1 (*DLK1*) is a maternally imprinted and paternally expressed gene that belongs to the *DLK1-DIO3* gene cluster on chromosome 14q34. It was initially identified as a non-canonical Notch ligand with established roles during development and supportive functions in several aggressive cancers [27]. Elevated DLK1 expression has been documented across a spectrum of cancers affecting various organ systems, including the endocrine system, gastrointestinal tract, lungs, liver, kidneys, brain, breast, sarcomas, and both pediatric and adult blood cancers. Its increased expression often correlates with advanced disease stages and is associated with worse outcomes in terms of progression-free survival (PFS), recurrence-free survival (RFS), and overall survival (OS). Moreover, in recent years it has been suggested that DLK1 plays a fundamental role in regulating cancer cell plasticity toward a less differentiated, more stem-like phenotype that may confer increased aggressiveness and therapeutic resistance and disease relapse across diverse malignancies [28,29,30].

Cancer stem cells (CSCs) are a small yet essential subpopulation within tumors, characterized by their ability to self-renew, differentiate, and initiate tumor growth. The existence of thyroid CSCs has been confirmed through in vitro generation of thyrospheres and in vivo tumor studies. Several markers, including CD44, CD133, and ALDH1, have been proposed to identify thyroid CSCs [30,31,32,33]. In medullary thyroid carcinoma (MTC), emerging evidence underscores the role of CSCs in driving tumor heterogeneity and recurrence. Therefore, targeting CSC-specific pathways may lead to more effective therapeutic strategies, ultimately reducing recurrence rates and improving long-term patient outcomes.

In this study, we conducted a meticulous examination of the expression of a range of well-established stemness markers, alongside potential stem cell markers identified in the MTC literature [20,21,31]. Given that multicellular spheroid generation is often used as an experimental model to measure the self-renewal, multidrug resistance, and multipotent nature of the cancer stem cell (CSC) subpopulation within a tumor or cancer cell line, we further investigated the ability of these cell lines to form multicellular spheroids and express multidrug resistance proteins. We next investigated DLK1 expression in medullary and non-medullary thyroid carcinoma cells, isolated subpopulations of MTC cells expressing DLK1 (DLK1+) and those lacking DLK1 expression (DLK1−) and subjected them to a detailed characterization of their expression of stem cell markers.

## 2. Results

### 2.1. Distinct Molecular Signatures of Stem Cell Markers in MTC Cell Lines

**Figure 1:**
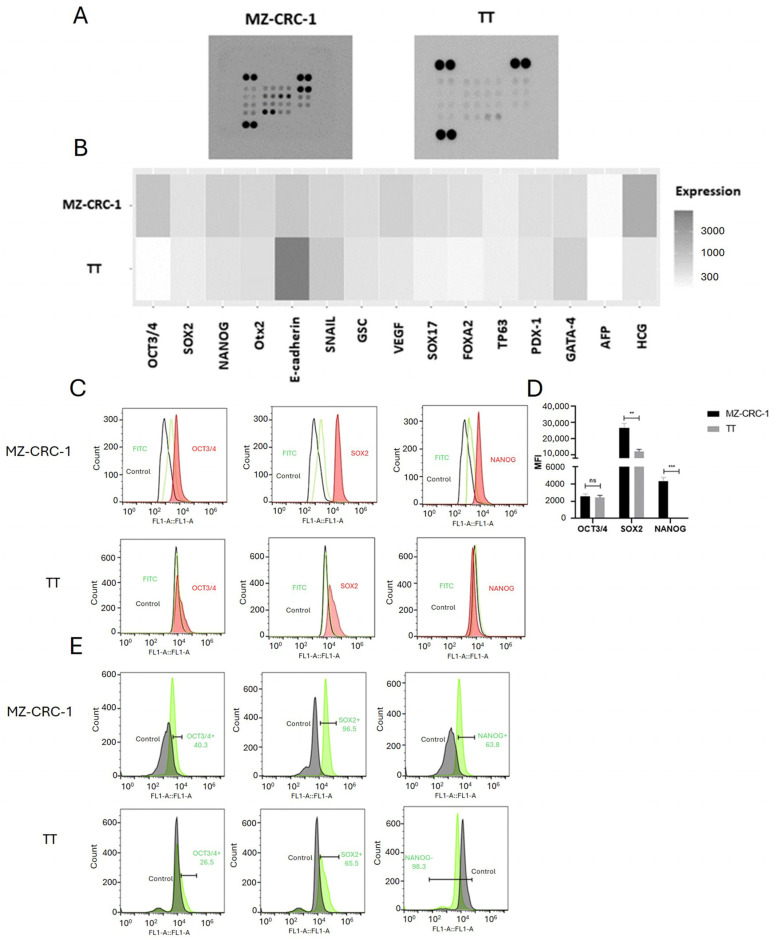
The figure provides a comprehensive analysis of stem cell markers in medullary thyroid carcinoma (MTC) cell lines. (**A**) shows results from antibody array membrane analysis, while (**B**) displays a heatmap illustrating the differential expression of 15 analyzed proteins. Notably, the MZ-CRC-1 cell line, characterized by the RET p.M918T variant, shows elevated expression for most proteins associated with the stem cell phenotype compared to the TT cell line, characterized by the RET p.C634W variant. (**C**) FC was employed to assess the expression of recognized stem cell markers (OCT3/4, SOX2, NANOG) in MZ-CRC-1 and TT cell lines. Representative histograms illustrate the red peak indicating target protein expression, distinctly separated from the green peak (secondary antibody) and black peak (negative control). (**D**) For FC analysis, two independent experiments were conducted in triplicate. About 10,000 events were recorded and reported as median fluorescence intensity (MFI). (**E**) FC indicates the percentage of MZ-CRC-1 and TT cells positive for OCT3/4 (40.3%, 26.5%), SOX2 (96.5%, 60.5%), and NANOG (63.8%, 1.7%). Statistical significance was determined using unpaired *t*-tests and denoted as follows: >0.05 (ns), ** *p* ≤ 0.01, and *** *p* ≤ 0.001.

The stemness profile of two MTC cell lines was obtained using a semi-quantitative antibody-based array for the parallel detection of 15 established stem cell markers (Table 1). Across both MZ-CRC-1 cells and TT cells, most markers associated with pluripotency, cell self-renewal, the maintenance of stem cell properties, and angiogenesis exhibited elevated expression levels. Remarkably, the expression levels of OCT3/4 (*p* = 0.009), SOX2 (*p* = 0.0428), NANOG (*p* = 0.031), OTX2 (*p* = 0.0423), VEGF (*p* = 0.0139), SOX17 (*p* = 0.0282) and PDX-1 (*p* = 0.0247) were significantly higher in MZ-CRC-1 cells compared to TT cells (Figure 1A,B).

Unpredictably, the expression of hCG, primarily produced by the placenta during pregnancy, was notably higher in MZ-CRC-1 cells compared to TT cells (*p* = 0.0035). On the other hand, the expression of SNAIL (*p* = 0.0059) and E-Cadherin (*p* = 0.0017) were significantly higher in TT cells compared to MZ-CRC-1 cells. The expression levels of GSC, FOXA2, TP63, GATA-4, and AFP were not found to differ significantly between the two cell lines (Figure 1B, Table 1). These results underscore distinct molecular signatures and potential functional differences between the MTC cell lines.

### 2.2. Validation Analysis Confirmed Higher Expression of Stemness Markers in MZ-CRC-1 Cells

To validate the findings from the proteomic profile array, we investigated the expression of the widely recognized stemness markers OCT3/4, SOX2, and NANOG using flow cytometry (FC). The histograms from the FC analysis illustrate the expression levels of these pluripotency factors in both MZ-CRC-1 and TT cells. The rightward shift of the red peaks (staining) in the histograms demonstrates the expression of OCT3/4, SOX2, and NANOG. This shift clearly distinguishes the signal from the secondary antibody (green peak) and negative control (black peak), confirming specific binding to the target proteins. The negative control cells were treated solely with the secondary antibody, as indicated by the green peak (Figure 1C). From examination of the histogram bar graphs, we observe a significant increase in the MFI of SOX2 (*p* = 0.001) and NANOG (*p* < 0.0001) in MZ-CRC-1 cells compared to TT cells (Figure 1D). Although the MFI of OCT3/4 was slightly higher in MZ-CRC-1 cells, this difference did not achieve statistical significance relative to TT cells. Importantly, the percentage of positive cells for these markers was significantly higher in MZ-CRC-1 compared to TT cells: OCT3/4 (40.3% vs. 26.5%), SOX2 (96.5% vs. 60.5%), and NANOG (63.8% vs. 1.7%) (Figure 1E). These findings underscore the heightened stemness profile exhibited by MZ-CRC-1 cells.

### 2.3. The Expression of Hypothetical Thyroid Cancer Stem Cells in MTC Cell Lines

**Figure 2:**
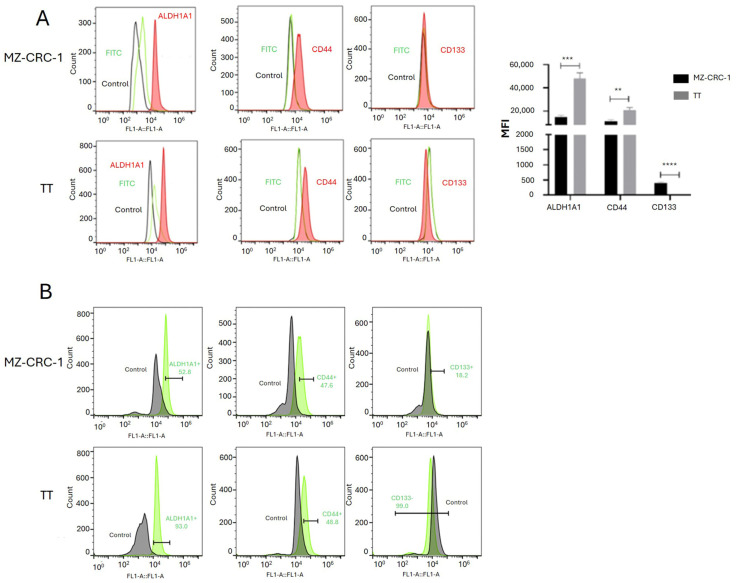
The figure displays the FC analysis of ALDH1A1, CD44, and CD133. (**A**) shows representative histograms of ALDH1A1, CD44, and CD133 expression, with the red peak indicating target protein expression. This distinct shift separates it from the secondary antibody signal (green peak) and the negative control (black peak). For FC analysis, two independent experiments were conducted in triplicate. About 10,000 events were recorded and reported as median fluorescence intensity (MFI). (**B**) shows histograms illustrating the proportion of cells expressing the target protein (green peak), distinctly separated from the negative control (black peak). The percentage of MZ-CRC-1 and TT cells positive for ALDH1A1 (52.1%, 93%), CD44 (47.6%, 48.8%), and CD133 (18.2%, 1%) is shown. Statistical significance was determined using unpaired *t*-tests and denoted as follows: ** *p* ≤ 0.01, *** *p* ≤ 0.001, and **** *p* ≤ 0.0001.

Flow cytometry was employed to assess the expression of putative thyroid cancer stem cell markers CD44 and CD133, as well as ALDH1A1, in MTC cell lines. Our analysis revealed that both MZ-CRC-1 and TT cells expressed ALDH1A1 and CD44 markers, whereas CD133 expression was specifically detected in MZ-CRC-1 cells (Figure 2A). As illustrated in the histogram bar graphs, the MFI levels of ALDH1A1 (*p* = 0.0003) and CD44 (*p* = 0.0022) were higher in TT cells compared to MZ-CRC-1 cells. Conversely, CD133 exhibited higher expression levels in MZ-CRC-1 cells (*p* < 0.0001). Moreover, the proportion of cells expressing ALDH1A1 (52.1% in MZ-CRC-1 vs. 93% in TT) and CD44 (47.6% in MZ-CRC-1 vs. 48.8% in TT) was higher in TT cells. Conversely, the proportion of cells expressing CD133 was greater in MZ-CRC-1 cells (18.2% vs. 1%) (Figure 2B). These findings highlight the distinct expression patterns and proportions of cells expressing these potential biomarkers in the two MTC cell lines.

### 2.4. DLK1 Is Specifically Expressed in MTC Cell Lines

**Figure 3:**
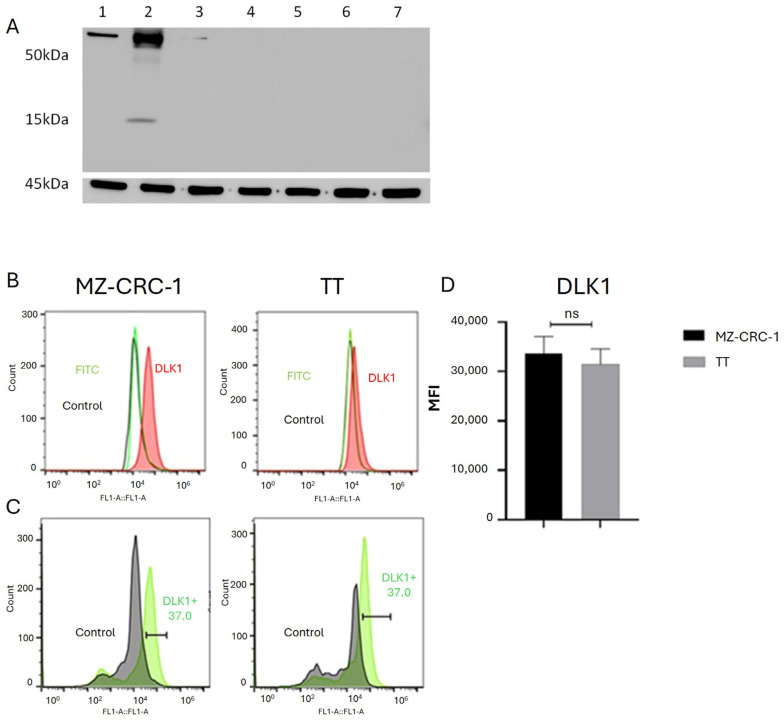
(**A**) The upper panel presents representative Western blot results showing DLK1 expression in TT (line 1) and MZ-CRC-1 (line 2) cells, as well as in non-medullary thyroid cell lines FTC133, BCPAP, XTC.UC1, KTC2, and 8505 (lines 3–7). β-actin was used as a loading control in the lower panel. (**B**) Representative histograms from FC analysis depict DLK1 expression in MZ-CRC-1 and TT cells. The red peak in the histograms indicates DLK1 protein expression, clearly distinguishable from the secondary antibody signal (green peak) and the negative control (black peak). (**C**) illustrates the proportion of cells expressing DLK1 in MZ-CRC-1 (37%) and TT cells (34.5%). (**D**) FC analysis was performed in triplicate across two independent experiments, and median fluorescence intensity (MFI) was reported. Statistical significance was assessed using unpaired *t*-tests and is denoted as >0.05 (ns).

DLK1, a surface protein belonging to the NOTCH noncanonical ligand family, is co-expressed with stem cell markers in various human cancers and is therefore implicated in regulating stem cell properties. This study investigates DLK1 expression in two medullary thyroid carcinoma cells (MZ-CRC-1 and TT cells) and five non-medullary thyroid carcinoma cell lines (FTC133, BCPAP, XTC.UC1, KTC2, and 8505). Figure 3A shows that DLK1 expression was significantly higher in the MZ-CRC-1 cell line (line 2) compared to the expression observed in TT cell line (line 1). In contrast, no DLK1 expression was detected in the follicular-derived cell lines (lines 3–7), reinforcing its potential pathogenic role in MTC. Moreover, in the MZ-CRC-1 cell line, two isoforms of DLK1 were identified: the full-length isoform (50–55 kDa) and a smaller isoform of approximately 15 kDa. Β-actin (45 kDa) was used as a loading control (Figure 3A).

Flow cytometry analysis confirmed DLK1 expression in both MZ-CRC-1 and TT cells (Figure 3B), indicating that the membrane-bound isoform of DLK1 is present in both MTC cell lines and may contribute to maintaining the stem cell phenotype. Representative histograms show that DLK1 expression was higher in MZ-CRC-1 cells compared to TT cells (Figure 3B), with a slightly greater proportion of DLK1 positive cells in MZ-CRC-1 (37%) than in TT cells (34.5%) (Figure 3C), though this difference was not statistically significant. For FC analysis, two independent experiments were conducted in triplicate. About 10,000 events were recorded and reported as median fluorescence intensity (MFI) (Figure 3D).

### 2.5. Expression of Multidrug Resistance Protein in Medullary Thyroid Carcinoma Cells 

**Figure 4:**
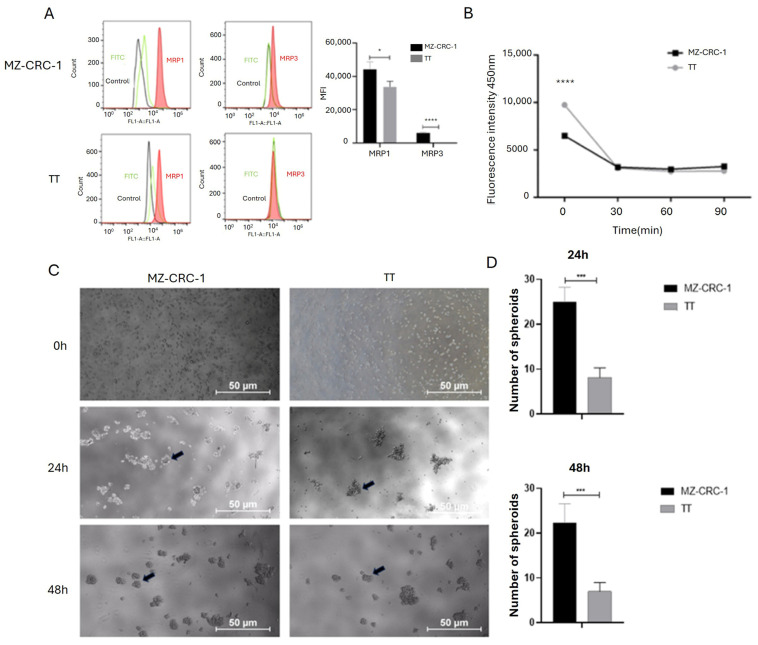
The figure depicts the FC analysis of MRP1 and MRP3 and spheroid formation in MTC cells. (**A**) Representative histograms from FC analysis illustrate the red peak indicating target protein expression, distinctly separated from the green peak (secondary antibody) and black peak (negative control). FC analysis was performed in triplicate across two independent experiments, and median fluorescence intensity (MFI) was reported. (**B**) illustrates the concentration of Hoechst 33342 dye in cell supernatant at specified time points. Two independent experiments were conducted in triplicate. (**C**) The images illustrate representative examples of spheroid formation in MTC cells captured at distinct time points (24 h and 48 h after plating). The arrow highlights one of the multiple spheroids visible in the image. (**D**) Two independent experiments were conducted in triplicate. The total numbers of multicellular spheroids at each time point are graphically represented. Statistical significance was assessed using an unpaired *t*-test (* *p* < 0.05, *** *p* < 0.001, **** *p* < 0.0001).

As CSCs are associated with resistance to conventional therapies, primarily due to the overexpression of multidrug resistance proteins (MRPs), which leads to reduced concentrations of chemotherapeutic drugs within cells, we assessed the expression levels of MRP1 and MRP3 proteins in MTC cells using flow cytometry. Upon examination of the histogram bar graphs, we observed that the MZ-CRC-1 cell line expresses higher levels of MRP1 (*p* = 0.0302) and MRP3 (*p* < 0.0001) compared to TT cells (Figure 4A). These findings suggest that the MZ-CRC-1 cell line may have a greater potential for developing drug resistance and maintaining a stem cell phenotype compared to TT cells.

### 2.6. Enhanced Spheroid Generation Ability in MZ-CRC-1 Cells

As CSCs possess the ability to efflux the fluorescent dye Hoechst 3342, often mediated by multidrug resistance efflux pumps, we conducted an exclusion assay in both MTC cell lines. As shown in Figure 4B, the intracellular accumulation of the dye is significantly lower in MZ-CRC-1 than in TT cells (*p* < 0.0001) (Figure 4B). These findings align with the observed expression levels of the MRP1 and MRP3 efflux pumps in MZ-CRC-1 and TT cells

Previous studies on medullary thyroid carcinoma (MTC) have indicated that the MZ-CRC-1 cell line, harboring the *p*.M918T RET mutation, exhibits greater numbers of CD133+ cells and enhanced sphere-forming ability compared to the TT cell line. To confirm whether these two cell lines (TT and MZ-CRC-1) differ in their ability to grow under adherence-free conditions, we conducted the multicellular spheroid generation assay. MZ-CRC-1 and TT cell lines were seeded into plates with serum-free medium. Spheroid growth was monitored for 24 and 48 h using an inverted phase-contrast microscope. Representative images of the spheroids are shown in Figure 4C. While both cell lines formed spheroids, indicating CSC presence and stemness marker expression, notable differences were observed between them regarding spheroid number and size. Specifically, MZ-CRC-1 demonstrated a higher number of spheroid formations compared to TT cells at both time points (*p* < 0.001). The results, presented as mean ± standard deviation, are shown in Figure 4D. Regarding the size of spheroids, no significant difference was observed between MZ-CRC-1 and TT cells at 48 h (Figure S1).

### 2.7. Cell Sorting and Characterization of DLK1-Positive Cells

To investigate the association between DLK1 and stem cell phenotype, we isolated DLK1 positive (DLK1+) and DLK1 negative (DLK1) subpopulations from the TT and MZ-CRC-1 cell lines via flow cytometry.

**Figure 5:**
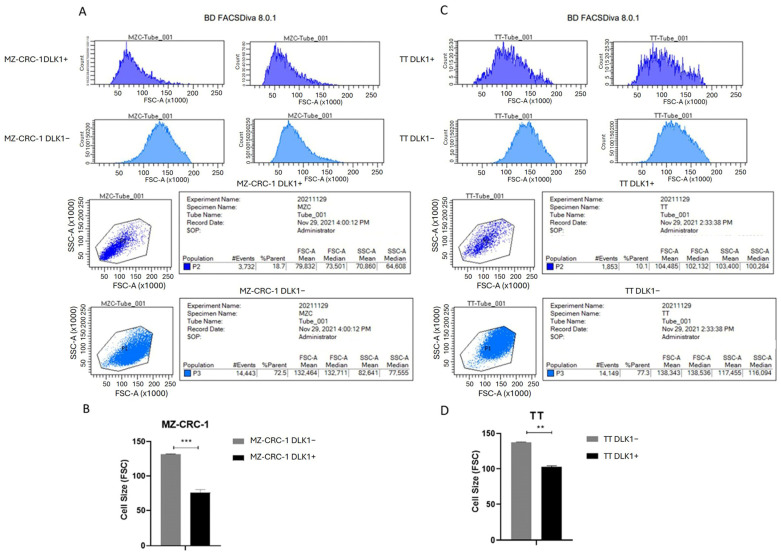
The figures illustrates the forward- (FCS) and side- (SSC) scatter density plots of MZ-CRC-1 (**A**) and TT cell (**C**) lines sorted by DLK1 expression, either positive (DLK1+) or negative (DLK1−) (light blue). Size (FSC) and granularity (SSC) were smaller in DLK1+ (dark Blue) than (DLK1−) (light blue) cells. The FSC versus SSC plot is showed. (**B**,**D**) The bar chart presents the mean cell size of each population, highlighting that the DLK1+ subpopulation (P2) exhibits significantly smaller size compared to DLK1− subpopulation (P3) (** *p* ≤ 0.01, *** *p* < 0.001).

Since smaller size can be a key parameter for isolating and studying stem-cell-like populations, we first compared the cell sizes and granularity of DLK1+ and DLK1− subpopulations. The light-scattering parameters of the cells increase with cell size and complexity. Here, we demonstrated that forward scatter area (indicating cell size) and side-scatter area (indicating granularity) were lower in DLK1+ cells (dark blue) compared to DLK1− cells (light blue) (*p* < 0.01) in both MZ-CRC-1 and TT cells. Forward-scatter area (FSC-A) versus side-scatter area (SSC-A) plot is showed in MZ-CRC-1 and TT cells (Figure 5A,C). Notably, this difference was more pronounced in the MZ-CRC-1 cells (*p* < 0.001) (Figure 5B,D).

**Figure 6:**
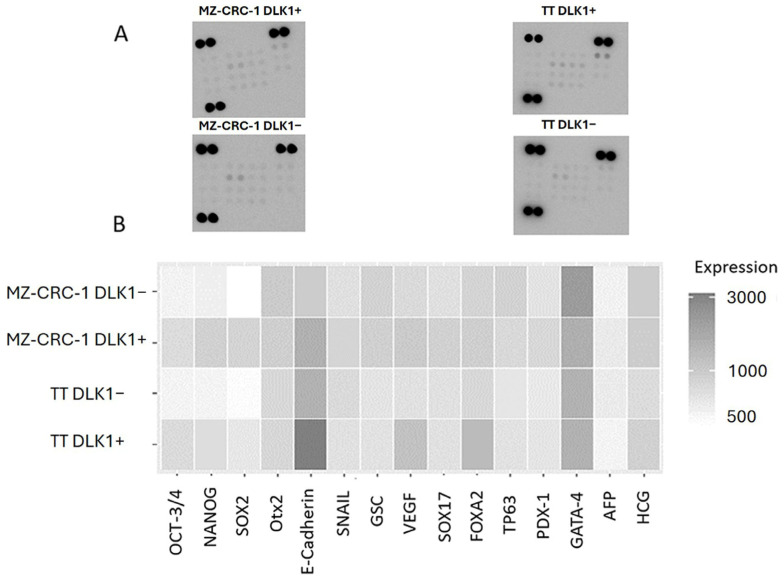
(**A**) presents results from an antibody array membrane analysis comparing DLK1-positive (DLK1+) and DLK1negative (DLK1−) subpopulations from the MZ-CRC-1 and TT cell lines. (**B**) displays a heatmap illustrating the differential expression of 15 analyzed proteins between the DLK1+ and DLK1− subpopulations (see Table 2).

We then evaluated the expression levels of established CSC markers in these isolated cell populations using an antibody-based array (Figure S2). DLK1+ subpopulations from both cell lines displayed heightened expression levels of pluripotency markers, such as SOX2, OCT3/4, and NANOG (*p* < 0.05), underscoring the association between DLK1 and the preservation of the CSC phenotype in MTC cell lines (Table 2 and Figure 6). Furthermore, besides the core transcription factors, other elements contributing to self-renewal capacity and pluripotency were also expressed at elevated levels in DLK1+ cells, indicating a link between DLK1 expression and the stem cell phenotype. A few genes showed reduced expression in DLK1+ cells, such as GATA-4 (Figure 6).

**Figure 7:**
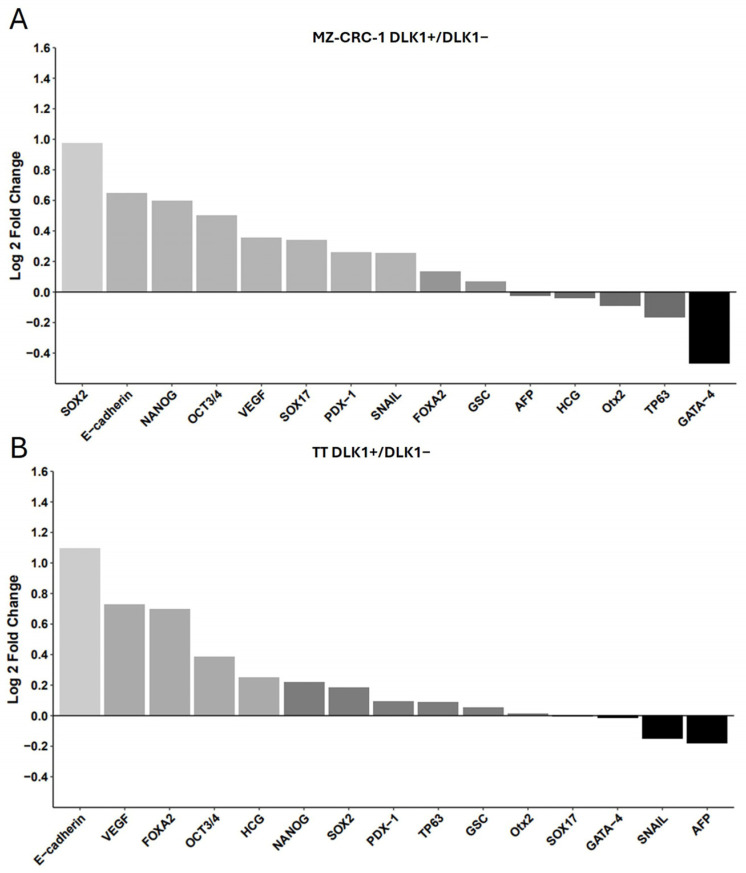
This figure presents the analysis of stem cell markers in DLK1-positive (DLK1+) compared to DLK1-negative (DLK1−) subpopulations in both MZ-CRC-1 (**A**) and TT (**B**) cell lines. The data presented in Table 2 were log-transformed, and a histogram was generated using RStudio software to compare the relative expression levels between DLK1+ and DLK1− cells in both cell lines. The bar chart depicts the fold change (log2) on the y-axis, highlighting 15 stemness markers. Proteins significantly upregulated in the DLK1+ subpopulation are represented in light gray, while those showing downregulation are depicted in darker gray or black for each cell line.

To better visualize the differences in expression, the data were log-transformed to maintain proportionality in both positive and negative fold changes. To improve the clarity and accuracy of visualizations, a histogram was generated using RStudio software (version 2022.07.2+576) to compare the relative expression levels between DLK1+ and DLK1− cells in both cell lines (Figure 7).

## 3. Discussion

Although MTC is less prevalent compared to differentiated thyroid carcinomas, its prognosis is usually worse. Approximately 50% of patients experience cervical lymph node metastasis involvement, while about 30% develop distant metastasis, affecting organs such as the liver, lungs, bones, and occasionally the skin and brain [32].

The primary treatment for MTC involves total thyroidectomy with neck dissection for lymph nodes. Due to their ineligibility for radioactive iodine therapy, patients with progressive disease may undergo radiotherapy or systemic therapy, which encompasses cytotoxic chemotherapy, RET-selective inhibitors, multi-tyrosine kinase inhibitors (TKIs), or immunotherapy [33]. Although response rates vary, systemic chemotherapy typically yields partial or transient responses. Among cytotoxic drugs, doxorubicin, either as a monotherapy or combined with cisplatin, is the most commonly employed in MTC patients [34]. However, cytotoxic chemotherapy, while capable of controlling tumor burden, is not considered the first-line therapy for patients with persistent or recurrent MTC due to its low efficacy. Instead, targeted therapies such as tyrosine kinase inhibitors (e.g., vandetanib and cabozantinib) are typically preferred as first-line options for managing persistent or recurrent MTC in many countries [32,33,34,35,36].

New selective RET inhibitors (e.g., selpercatinib or pralsetinib) have revolutionized the treatment of cancer and have been approved as a treatment option for cancers with *RET* gene alterations. While targeted therapies have demonstrated superior efficacy and tolerability when compared to conventional chemotherapy in treating MTC, they may not achieve the complete eradication of cancer cells. Regrettably, clinical responses for advanced MTC are often partial, and patients may eventually develop resistance to these drugs, leading to disease progression over time. Understanding the mechanisms of resistance is crucial for devising more effective and durable therapeutic strategies [37,38,39,40,41,42].

Emerging evidence suggests that drug resistance in cancers may arise from the survival of cancer cells displaying a stem cell phenotype. These cells (i.e., cancer stem cells) also play a critical role in tumor initiation and progression, as demonstrated by their ability to initiate new tumors when injected into animal models [43].

CSCs express canonical markers associated with self-renewal and pluripotency, including core proteins, drug efflux transporters, and surface markers such as CD44 and CD133 [20,21,27,44,45,46]. These molecular features are essential for tumor progression and metastasis, as they enhance the survival of CSCs and enable them to withstand conventional therapies. Moreover, CSCs exhibit the unique ability to form multicellular spheroids, potentially enabling their dissemination via the bloodstream or lymphatic circulation, thereby facilitating the development of distant metastases. This multifaceted role of CSCs underscores their pivotal significance in tumor biology and emphasizes the need for innovative therapeutic strategies targeting cancer progression and metastasis [47,48,49].

Consequently, several groups have suggested that CSC markers can be used to predict the prognosis of patients [50,51]. Also, there is evidence that CSC markers can distinguish CSCs from other cancer cells, enabling the identification of circulating CSCs before metastasis formation through liquid biopsy. These markers also hold potential as targets for innovative therapeutic approaches [52,53].

In the field of thyroid research, substantial experimental evidence has provided support to the hypothesis that CSCs are central in both the initiation of cancer and the formation of metastases [54,55,56,57,58,59,60,61,62,63]. The upregulation of cell surface markers such as CD44, CD133, and ALDH1A1 has been linked to a poorer prognosis and increased resistance to chemotherapy and radiotherapy in both differentiated (DTC) and undifferentiated thyroid carcinomas (UTC) [21,64,65]. However, there is limited information available regarding their role in MTC.

Previously, we identified a recurrent somatic copy number gain in the 14q32.2 region in MTC samples, which comprises the gene *DLK1*. Validation analysis showed that *DLK1* gain is correlated with increased protein expression, larger tumor size and advanced tumor stage (III and IV) [26]. Our findings, corroborated by others, indicate that the *DLK1* gene is frequently overexpressed in MTC [26,66].

*DLK1*, located on chromosome band 14q32.2 in humans, is an imprinted gene encoding secreted and membrane-bound isoforms. While its expression is widespread in various human tissues during embryonic development, in adults, it is predominantly restricted to (neuro)endocrine tissues and other immature stem/progenitor cells. Notably, *DLK1* exhibits elevated expression levels in numerous common malignancies, including liver, breast, brain, pancreas, colon, and lung cancers, as well as endocrine-related cancers such as ovarian tumors and pheochromocytoma. Indeed, *DLK1*’s elevated expression has been identified in pediatric cancers as well as aggressive and therapy-resistant adult cancers [28,67,68,69,70].

Recent studies suggest that drug resistance in cancers may stem from the survival of cancer cells exhibiting a stem cell phenotype, and DLK1 may play a crucial role in regulating and maintaining this phenotype [30,71].

In this study, to assess their stem cell-like properties, we characterized two MTC cell lines for the expression of 15 known stem cell markers. These lines harbor pathogenic variants in the two most common MEN2A- and MEN2B-associated codons (p.C634 and p.M918), enabling us to investigate the relationship between these RET variants and the stem cell phenotype. MZ-CRC-1 cells, which carry the pathogenic variant p.M918T, associated with MEN2B syndrome, exhibited higher expression levels of most canonical markers compared to TT cells, which possess the p.C634W variant linked to MEN2A.

The protein-array analysis revealed that most markers that are fundamental for the initiation and maintenance of pluripotent states were expressed in both cell lines, with MZ-CRC-1 exhibiting higher levels of most markers. This suggests that MZ-CRC-1 possesses a robust stem cell phenotype, characterized not only by the presence of key pluripotency factors such as OCT3/4, SOX2, and NANOG but also by additional markers that may enhance stemness. Validation of the canonical stem cell markers (OCT3/4, SOX2, NANOG) by flow cytometry confirmed their expression in both cell lines and increased expression in MZ-CRC-1 cells.

Subsequent assessment of CD44, CD133, ALDH1A1, multidrug resistance proteins (MRP1 and MRP3), and DLK1 using flow cytometry revealed distinct expression patterns. Specifically, CD133 and MRP proteins exhibited higher levels in MZ-CRC-1 cells, while CD44 and ALDH1A1 were elevated in TT cells.

Although both cell lines contain a subpopulation of cells with a cancer stem cell (CSC) phenotype that may influence tumor progression, aggressiveness, and drug resistance, there are slight differences in their expression profiles.

One potential hypothesis is that the observed differences may stem from the genetic background and biological behavior of the medullary thyroid carcinoma (MTC) cells. MZ-CRC-1 cells, which carry the p.M918T RET variant, exhibited higher expression levels of canonical markers, including CD133, DLK1, and multidrug resistance proteins (MRP), compared to TT cells, which harbor the less aggressive p.C634W RET variant. This disparity may be linked to the functional impact on tyrosine kinase activity. The p.M918T variant is located in the activation loop of the RET receptor tyrosine kinase, leading to constitutive activation of RET and heightened signaling activity, even in the absence of ligand binding. While the p.C634W variant also activates the RET pathway, its effect is generally less potent than that of p.M918T, resulting in a more moderate increase in RET signaling and a less aggressive tumor phenotype. However, further analysis is needed to validate this hypothesis.

Notably, Western blot analysis revealed that DLK1 was specifically identified in MTC cell lines, suggesting a distinct role for this protein in MTC. Contrary to the expectations from FC analysis, DLK1 exhibited significantly higher expression levels in MZ-CRC-1 cells compared to TT cells. These data suggest that the membrane-bound isoform of DLK1 is likely expressed at slightly different levels in these cell lines, as observed in the flow cytometry data. However, MZ-CRC-1 cells may also express additional spliced isoforms of DLK1, including the cleaved ligand, the cytoplasmic component resulting from cleavage, or the membrane-bound isoform.

Notably, MZ-CRC-1 cells also enhanced spheroid-forming ability compared to TT cells.

Further, our investigation involved sorting cells into DLK1-positive and -negative populations and assessing stem cell marker levels in both groups. The DLK1+ subpopulation in both cell lines expressed stemness markers, with notably higher expression in MZ-CRC-1 compared TT cells.

While this study uncovers novel insights, it is acknowledged that further investigations, including gene knockdown experiments and in vivo analyses, are needed to elucidate DLK1’s role comprehensively. We also acknowledge that besides somatic copy number alteration, other mechanisms may exert an influence on DLK1 expression.

## 4. Materials and Methods

### 4.1. Cell Culture

This study utilized two RET-driven MTC cell lines: MZ-CRC-1 (harboring p.M918T mutation) and TT (harboring p.C634W mutation). Additionally, five non-MTC carcinoma cell lines were included: FTC133 (lymph node metastasis from follicular thyroid carcinoma), B-CPAP (papillary thyroid carcinoma), and XTC.UC1 (oncocytic cell carcinoma), as well as 8505c and KTC-2 (undifferentiated thyroid carcinoma).

The TT cell line was obtained from the ATCC (Cat# CRL-1803), while the MZ-CRC-1 cell line was generously provided by Prof. Barry Nelkin from Johns Hopkins University. The XTC.UC1 cell line was kindly donated by Prof. Ian Ganly from Memorial Sloan Kettering Cancer Center (New York, NY, USA). The BCPAP (ACC 273) and 8505c (ACC 219) cell lines were purchased from Leibniz Institute DSMZ (Braunschweig, Germany). The FTC133 (CC 94060901) was purchased from Sigma. The TT cell line was cultured in F-12K medium supplemented with 10% FBS and 100 U/mL penicillin/streptomycin. The MZ-CRC-1 and XTC.UC1 cell lines were cultured in a 1:1 mixture of DMEM and Ham’s F12, supplemented with 10% FBS and 100 U/mL penicillin/streptomycin. FTC133 was cultured in 1:1 mixture of DMEM and Ham’s F12, supplemented with 10% FBS. The BCPAP and 8505c cell lines were grown in RPMI-1640 medium supplemented with 10% FBS and 100 U/mL penicillin/streptomycin. The KTC-2 cell line was grown in RPMI-1640 medium supplemented with 5% FBS and 100 U/mL penicillin/streptomycin. All cells were maintained at 37 °C in a 5% CO_2_ humidified atmosphere. To ensure the reliability and authenticity of the thyroid cancer cell lines used in our study, we conducted a panel of short tandem repeat (STR) profiling. This step was essential to mitigate concerns about potential cross-contamination and to maintain the integrity of our experimental results.

### 4.2. Protein Array Analysis

To explore the expression of known CSC markers in the TT and MZ-CRC-1 cell lines, protein extracts isolated from the cells underwent analysis using the antibody-based human pluripotent stem cell proteomic array (Cat # ARY010; R&D Systems, Minneapolis, MN, USA). This array simultaneously detects 15 established stem cell markers (Table 1 and Figure S2). Approximately 10^7^ MZ-CRC-1 and TT cells were used for protein lysate isolation, and quantification was carried out as previously described [72].

Nitrocellulose membranes, each spotted with 15 different antibodies in duplicate, were incubated with 300 μg of cellular extract for 16 h at 4 °C. After two washes, the membranes were incubated with streptavidin-HRP secondary antibody and revealed with a chemiluminescent reagent (R&D Systems). The intensity score of each duplicated spot was analyzed using the ImageQuant LAS4000 Analyzer (GE Healthcare, Chicago, IL, USA) and quantified using ImageQuant TL software Version 1.1 Built 1.1.0.75 (GE Healthcare), as described previously [72]. The average intensity signal of the duplicate spots was determined by subtracting the average background signal (negative control) from each spot, as per the manufacturer’s instructions. Fold changes were then calculated based on the average density values of each protein expressed in the MZ-CRC-1 cells, divided by the average density values obtained in the TT cells.

### 4.3. Flow Cytometry Analysis

FC analysis was employed for validation, assessing the expression of recognized stem cell markers (OCT3/4, SOX2, NANOG), immunophenotyping cells for the expression of both recognized stem cell markers (ALDH1A1, CD44, and CD133) and a hypothetical (DLK1) stem cell marker in the thyroid, and measuring cell size at the single-cell level. FC was also utilized to measure the expression of multidrug resistance markers MRP1 and MRP3.

To prepare for FC, cells were fixed with 4% paraformaldehyde, blocked with 5% BSA, and then incubated with the appropriate primary antibodies, as detailed in Table S1. Subsequently, cells were incubated with the secondary labeled antibody, goat anti-rabbit IgG-FITC (Thermo Fisher Scientific, Waltham, MA, USA), and washed in 1X PBS. Two independent experiments were performed, each performed in triplicate. For each flow cytometric analysis, 10,000 events were recorded and analyzed on a BD Accuri C6 flow cytometer with a filter in FL1 (BD Biosciences, Franklin Lakes, NJ, USA). The resulting files were exported and analyzed using FlowJo software version 9 (Tree Star Inc., Ashland, OR, USA).

Negative controls included cells without primary or secondary antibodies, as well as cells incubated solely with the secondary antibody in a blocking buffer. Statistical analyses were based on the median fluorescence intensity (MFI), with results presented as flow cytometry histograms and bar graphs, following the recommendations outlined in [73].

### 4.4. Hoechst 33342 Efflux Assay

To evaluate the cells’ ability to efflux the fluorescent dye Hoechst 33342, approximately 8 × 10^3^ cells per well were cultured in a 96-well plate and exposed to 5 µg/mL of Hoechst 33342 (Thermo Fisher Scientific) for approximately 90 min at 37 °C. Following the staining period, the cells were washed once with PBS, and the medium was replaced. The plate was then transferred to a SpectraMax Plus M3 microplate reader (Molecular Devices, San José, CA, USA), the blue fluorescence of Hoechst 33342 was excited at 350 nm, and its fluorescence was measured at 450 nm (Time 0). Subsequently, fluorescence readings were taken at 30, 60, and 90 min time points (cumulative time points). Two independent experiments were performed in triplicate. Statistical analysis was performed using GraphPad Prism software version 8.0.1, and the results were presented graphically to illustrate the efflux kinetics of Hoechst 33342 from the cells over the specified time intervals.

### 4.5. Generation of Spheroid Cultures

To confirm the ability of MTC cell lines to form multicellular spheroids [74], 2 × 10^4^ cells were cultured in serum-free medium in 24-well ultra-low attachment plates (Corning, NY, USA). The spheroid formation efficiency (SFE) was monitored using a Nikon Eclipse TE2000 inverted microscope system (Nikon, Melville, NY, USA) equipped with an optical system and TCapture software version 4.3.0.605, which enabled us to capture high-quality images. Images were captured at 0, 24, and 48 h post-seeding. The quantification of spheroids was conducted by counting the number and measuring the size of spheroids at specific time points. Spheroid counting was performed manually. The size was measured using the TCapture software, which allows for precise measurements of the spheroids’ radii. Two independent experiments were conducted, and each experiment was performed in triplicate.

### 4.6. Western Blot Analysis

Cell homogenates were prepared from two medullary and five non-medullary thyroid cell lines and incubated on ice in radioimmunoprecipitation (RIPA) buffer supplemented with protease and phosphatase inhibitor cocktails (Merck, NJ, USA). After centrifugation at 14,000 rpm for 10 min, protein concentration was quantified using the BCA protein assay kit (Pierce Biotechnology, Rockford, IL, USA). Fifty micrograms of reduced protein were loaded onto a 10% polyacrylamide gel. The proteins were then transferred onto a nitrocellulose membrane (Bio-Rad, Hercules, CA, USA) and blocked for 1 h in TBS with 0.1% Tween and 5% nonfat dry milk before probing with primary antibodies against DLK1 (Ab21682, 1:500) and β-actin (Cat #4967, Cell Signaling, Danvers, MA, USA, 1:1000) (Table S1). Primary antibodies were incubated overnight at 4 °C in blocking buffer, followed by washing with TBST (150 mM NaCl, 2.7 mM KCl, 25 mM Tris, 0.1% Tween). The membrane was then incubated with anti-rabbit horseradish peroxidase-conjugated secondary antibodies (Dako, Carpinteria, CA, USA, P0448, 1:10,000) (Table S1) for 1 h at room temperature. Detection was performed using Immobilon Western Chemiluminescent HRP Substrate (Millipore, Burlington, MA, USA) and visualized with an ImageQuant LAS 4000 imaging system (GE Healthcare).

### 4.7. Fluorescence-Activated Cell Sorting (FACS)

To isolate a subpopulation of MZ-CRC-1 and TT cells expressing DLK1, approximately 2 × 10^7^ cells/mL were blocked with 2% BSA, followed by incubation with the primary antibody targeting DLK1 for 1 h. Subsequently, cells were fluorescently labeled with a secondary antibody before being subjected to FACS analysis using the BD FACS Aria II system (BD Bioscience). The DLK1-positive (DLK1+) and DLK1-negative (DLK1−) subpopulations were collected and subjected to protein isolation. Subsequently, the isolated proteins were analyzed using the Proteome Profiler Human Pluripotent Stem Cell Array, as aforementioned. The obtained data were analyzed to determine differential protein expression patterns between the DLK1+ and DLK1− subpopulations.

### 4.8. Statistical Analysis

Protein array analysis involved calculating fold changes based on the average density values of each protein expressed in MZ-CRC-1 cells divided by those in TT cells. Flow cytometry utilized the MFI from 10,000 events, analyzed with an unpaired *t*-test. The Hoechst 33342 assay was conducted in two independent experiments, each performed in triplicate, and analyzed using a *t*-test. Spheroid formation was quantified by counting and measuring spheroid number and size using TCapture software, with statistical analysis performed using a *t*-test in GraphPad Prism software. Relative expression levels between DLK1+ and DLK1− cells in both cell lines were compared after log-transforming the data (Log2) to enhance normality, maintain proportionality in fold changes (both positive and negative), and improve interpretability. A histogram was generated using RStudio software to visualize these comparisons.

## 5. Conclusions

Our study reveals the characteristics of cancer stem cell subpopulations within medullary thyroid carcinoma (MTC) cell lines, highlighting the potential role of DLK1 in promoting the stemness phenotype. These findings enhance our understanding of the underlying mechanisms in MTC and underscore the importance of targeting cancer stem cell populations to develop more effective therapeutic strategies.

## Figures and Tables

**Table 1:** Expression of stem cell markers in medullary thyroid carcinoma cell lines.

Protein	Average Intensity in TT Cells	Average Intensity in MZ-CRC-1 Cells	Fold Change *	*p*-Value
OCT3/4	173	941	5.439306	0.009
SOX2	283	389	1.374558	0.0428
NANOG	398	786	1.974874	0.031
OTX2	452	570	1.261062	0.0423
E-Cadherin	7956	893	0.112242	0.0017
Snail	846	589	0.696217	0.0059
GSC	430	479	1.113953	NS
VEGF	339	662	1.952802	0.0139
SOX17	249	492	1.975904	0.0282
FOXA2	218	435	1.995413	NS
TP63	272	341	1.253676	NS
PDX-1	359	434	1.208914	0.0247
GATA-4	660	469	0.710606	NS
AFP	163	192	1.177914	NS
HCG	335	2048	6.113433	0.0035

* The fold change was calculated based on the average density value of each protein expressed in MZ-CRC-1 divided by the average density values of TT cells. NS, not significant.

**Table 2:** Expression of stem cell markers in medullary thyroid carcinoma cell lines, stratified by DLK1 status.

Protein	Average Intensity in TT DLK1− Cells	Average Intensity in TT DLK1+ Cells	*p*-Value	Average Intensity in MZ-CRC-1 DLK1− Cells	Average Intensity in MZ-CRC-1 DLK1+ Cells	*p*-Value
OCT-3/4	528	689	0.0255	544	771	0.0041
NANOG	628	731	0.0119	569	859	0.0042
SOX2	563	639	0.0241	440	864	0.013
OTX2	810	818	NS	995	936	NS
E-cadherin	1495	3194	0.038	985	1544	0.011
Snail	781	703	NS	708	844	0.0091
GSC	669	695	NS	884	926	NS
VEGF	699	1156	0.0033	783	1002	NS
SOX17	703	700	NS	716	907	NS
FOXA2	803	1302	0.0245	856	941	NS
TP63	650	691	NS	887	792	NS
PDX-1	618	660	NS	679	811	NS
GATA-4	1511	1495	NS	2192	1587	0.0105
AFP	603	533	NS	619	608	NS
HCG	757	901	NS	988	962	NS

NS, not significant.

## Data Availability

The datasets generated during the current study are available from the corresponding author upon reasonable request.

## Supplementary Materials

The following supporting information can be downloaded at: https://www.mdpi.com/article/10.3390/ijms252211924/s1.
