# Supplementary material for: DLK1 Is Associated with Stemness Phenotype in Medullary Thyroid Carcinoma Cell Lines
Source: Int J Mol Sci. 2024 Nov 6;25(22):11924. doi: 10.3390/ijms252211924 (PMC11594232; doi:10.3390/ijms252211924)
Supplement: Supplementary file 1 [file ijms-25-11924-s001.zip › Legend of Supplementary Figures .pdf]

Legend of Supplementary Figures

Figure S1. Spheroid size in MTC cells is at 48h after plating. Two independent experiments were conducted in triplicate. Statistical analysis using an unpaired t-test revealed a significant difference (* $p < 0.05$)

Figure S2. (A) Human Pluripotent Stem Cell Array Spots (B) Reference table of coordinates for the Human Pluripotent Stem Cell Array.
