# Supplementary material for: DLK1 Is Associated with Stemness Phenotype in Medullary Thyroid Carcinoma Cell Lines
Source: Int J Mol Sci. 2024 Nov 6;25(22):11924. doi: 10.3390/ijms252211924 (PMC11594232; doi:10.3390/ijms252211924)
Supplement: Supplementary file 1 [file ijms-25-11924-s001.zip › Table S1.pdf]

Table S1 - List of antibodies used in flow cytometry and Western blot analysis

Antibody	m/pAb	Species	Manufacturer	Reference	Final Dilution
SOX2	mAb	Mouse	Abcam	ab79351	1:100
OCT3/4	mAb	Rabbit	Abcam	ab181557	1:100
ALDH1A1	pAb	Goat	Abcam	ab9883	1:200
CD44	pAb	Rabbit	Abcam	ab157197	1:100
CD133	pAb	Rabbit	Abcam	ab16518	1:100
MRP1	mAb	Mouse	Sta. Cruz	sc-365635	1:100
MRP3	pAb	Rabbit	Sta. Cruz	sc-5774	1:100
DLK1	pAb	Rabbit	Abcam	ab21682	1:100
β-actin	pAb	Rabbit	Cell Signalling	#4967	1:10.00
