# Supplementary figures and images for: DLK1 Is Associated with Stemness Phenotype in Medullary Thyroid Carcinoma Cell Lines
Source: Int J Mol Sci. 2024 Nov 6;25(22):11924. doi: 10.3390/ijms252211924 (PMC11594232; doi:10.3390/ijms252211924)

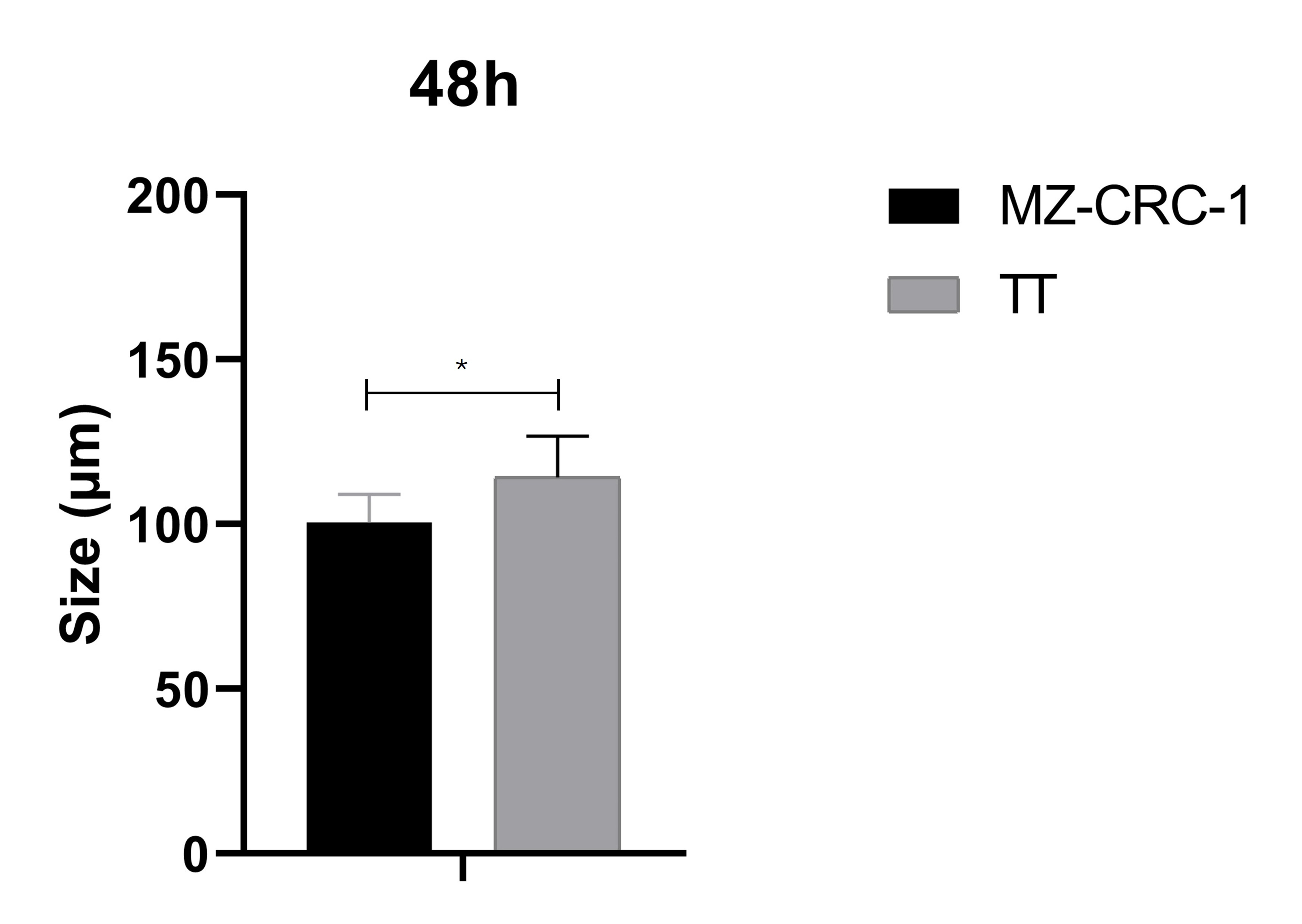

Supplement: Supplementary file 1 [file ijms-25-11924-s001.zip › Figure S1.jpg]

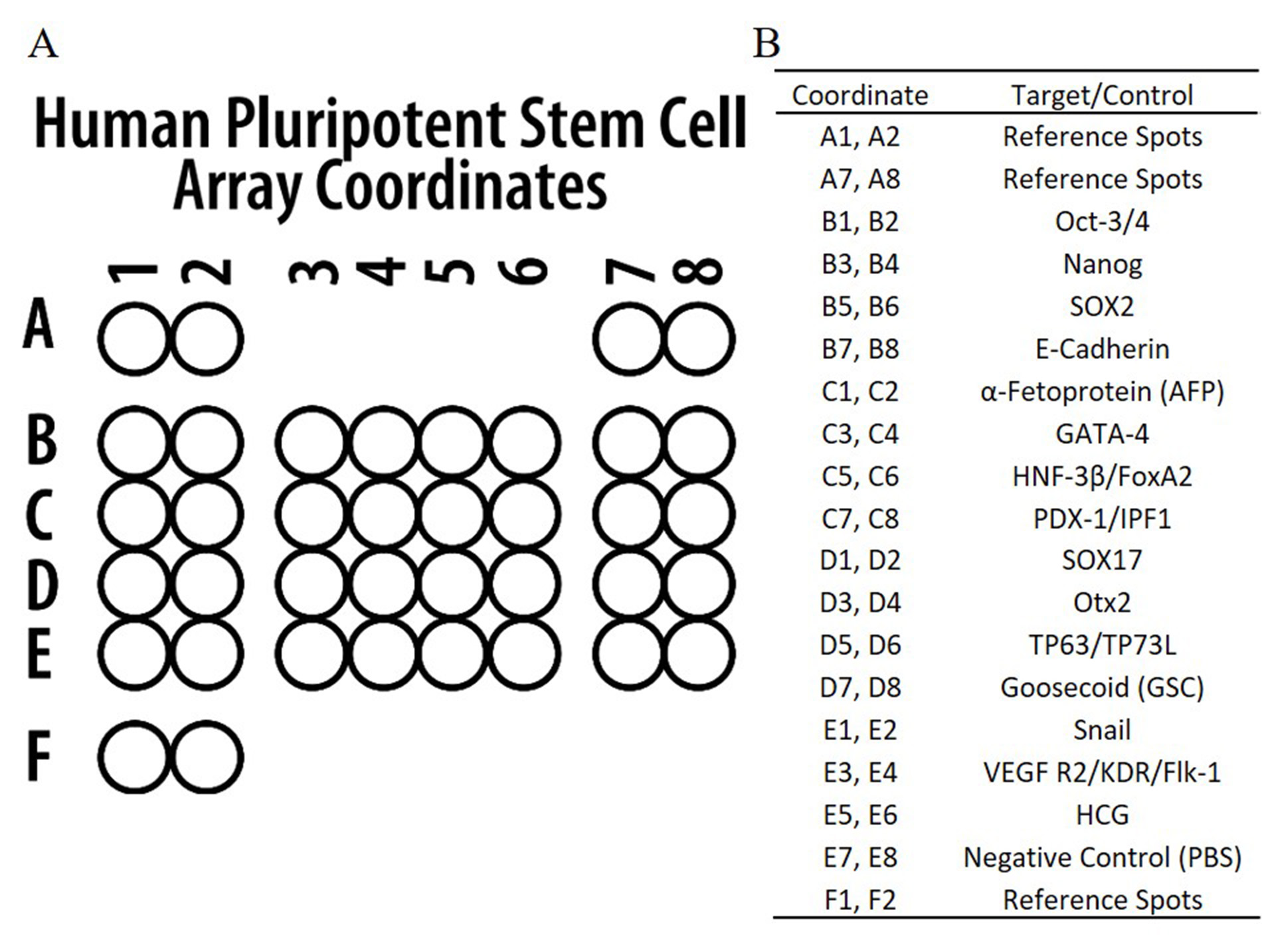

Supplement: Supplementary file 1 [file ijms-25-11924-s001.zip › Figure S2.jpg]
